# Rapid Data Analytics to Relate Sugarcane Aphid [(*Melanaphis sacchari* (Zehntner)] Population and Damage on Sorghum (*Sorghum bicolor* (L.) Moench)

**DOI:** 10.1038/s41598-018-36815-0

**Published:** 2019-01-23

**Authors:** Minori Uchimiya, Joseph E. Knoll

**Affiliations:** 1 USDA-ARS Southern Regional Research Center, 1100 Robert E. Lee Boulevard, New Orleans, LA 70124 USA; 2 USDA-ARS Crop Genetics and Breeding Research Unit, 115 Coastal Way, Tifton, GA 31793 USA

## Abstract

Sugarcane aphid [(*Melanaphis sacchari* (Zehntner)] emerged in the United States in 2013 as a new pest infesting sorghum (*Sorghum bicolor* (L.) Moench). Aphid population and plant damage are assessed by field scouting with mean comparison tests or repeated regression analysis. Because of inherently large replication errors from the field and interactions between treatments, new data analytics are needed to rapidly visualize the pest emergence trend and its impact on plant damage. This study utilized variable importance in the projection (VIP) and regression vector statistics of partial least squares (PLS) modeling to deduce directional relationships between aphid population and leaf damage from biweekly field monitoring (independent variable) and chemical composition (dependent variable) of 24 sweet sorghum cultivars. Regardless of environment, aphid population increase preceded the maximum damage rating. Greater damage rating at earlier growth stage in 2015 than 2016 led to an overall higher damage rating in 2015 than 2016. This trend in damage coincided with higher concentrations of trans-aconitic acid and polyphenolic secondary products in stem juice in 2016 than 2015, at the expense of primary sugar production. Developed rapid data analytics could be extended to link phenotypes to perturbation parameters (e.g., cultivar and growth stage), enabling integrated pest management.

## Introduction

Sorghum (*Sorghum bicolor* (L.) Moench) is a marginal land crop having a unique ability to grow on semi-arid soils^1^. The Renewable Fuel Standard Program of the U.S. Environmental Protection Agency recently approved a new pathway to produce biodiesel, jet fuel, and other renewable oil products from spent sorghum grain^2^. The extractable stem juice of “sweet” sorghum varieties offers additional benefits. Sweet sorghum juice is composed of fermentable sugars and chemical feedstocks (carboxylate and polyphenolic secondary products). Both primary and secondary products could be used to produce high market value specialty bio-based products including plastics^3^, green pesticides^4^, nematicides^5^, and antioxidant food additives^6^. In addition, acrylonitrile^7^ and other essential chemical feedstocks for industrial fibers, resins, and rubbers could be produced renewably from the microbial fermentation of stem sugars.

In 2013^8^, sugarcane aphid [(*Melanaphis sacchari* (Zehntner)] emerged as a new pest infesting sorghum in the United States, and has since become a perennial pest affecting much of the sorghum growing regions in the country^9^. Aphid population size, plant growth stage, and host plant resistance or tolerance determine the intensity of damage to sorghum^10^. Although resistant varieties are available commercially and through germplasm banks^8,11–15^, the underlying mechanisms causing aphid resistance or tolerance are largely unknown^9^.

Currently, field assessment of aphid infestation of sorghum, or other crops like wheat (*Triticum aestivum* L.), relies on manual scouting for aphid population counts and visual damage ratings^9–13^. Mean comparisons and means separation tests (ANOVA with Tukey’s honestly significant difference (HSD) test) of aphid density or damage rating are the primary methods used to interpret the effects of growth stage, insecticide treatment, and seasonal variation^8,10,16^. Alternatively, stepwise regression analysis is used to compare resistance among different sorghum varieties^12^ and to account for the spatial distribution of aphids^9^. Remote sensing^17–19^ and rapid data analytics are emerging as alternatives to quantify aphid population and damage with less bias than visual scoring. One available remote sensing study^17^ indirectly measured aphid-induced leaf damage through the changes in absorbance/reflectance at the wavelength ranges of chlorophyll.

Measured aphid density has inherently large replication errors that, if reported, span orders of magnitude^12^. This large error originates from the spatial variations controlled by the rates of departure and landing by alates (winged aphids)^9,20^. Aphids induce secondary damage to infested sorghum through the production of honeydew and subsequent saprophytic fungal growth (sooty mold)^21^. As a result, observable aphid population density may not directly relate to the crop damage scoring results. Composite aphid density and stepwise regression analysis have a limited ability to (1) deduce the relationships between population and damage and (2) evaluate treatment effects exhibiting interactions^10^. New analytics are in demand to rapidly and objectively interpret (1) aphid population and damage time courses (from planting to harvest) having large replication errors, (2) relationships between aphid population and crop damage, and (3) influence of host genotypes and environmental factors on (1) and (2). Our previous report developed partial least squares (PLS) calibration and prediction models based on UV/visible spectra of sweet sorghum juice and bagasse^22^. Spectra for juice samples were used to predict trans-aconitic acid concentration, while spectra from the methanol extract of bagasse were used to predict the relative concentrations of polyphenol-like fluorescent fingerprints^22^. Both phenolics^21,23,24^ and trans-aconitic acid^25^ have been proposed to act as defensive phytochemicals or phytohormonal signals upon leaf damage by aphids and other plant-feeding arthropods.

As described in detail elsewhere^26,27^, PLS constructs an inverse least squares model using a given number of components to predict a dependent variable (y) from a set of independent variables (X). Partial least squares offers several advantages over stepwise regression methods. First, when independent variables are collinear and contain random noise, variable importance in the projection (VIP) scores can be used to detect the variable range having a nonzero regression vector. The regression vector is the “slope” between the dependent and independent variables used to deduce their proportional or counter-proportional relationships^26^. The VIP scores estimate the importance of each variable in the projection used in the PLS model^26^. As a result, VIP and regression coefficient statistics embedded in PLS^28,29^ are used to visualize a subset of independent variables controlling the variation in response in diverse disciplines including plant ecology^30^, hyperspectral imaging^31^, and metabolomics^32^. For example, Luedeling *et al*.^33,34^, reported a series of ecological studies utilizing a threshold VIP value (above 0.8) to determine the atmospheric temperature (X) influencing the flowering dates (y) of apricot and peach (*Prunus* spp.) trees over decades. The regression coefficient was used to interpret the directional relationships (positive or negative slope between X and y) within temperature range and flowering dates meeting the cutoff criteria (VIP > 0.8)^33,34^.

In summary, variable selection by PLS is a useful method to reduce noise (besides Ridge or Lasso regression) and other data redundancy^35^, especially when bias-variance tradeoff leads to overfitting^30,36^. A threshold value of VIP, regression coefficient, or loading weight can be set to classify meaningful ranges of independent variables^29^. Consequently, PLS parameters highlight the data range where the variation in an independent variable is correlated with a dependent variable, and their directional relationships^34^. The objective of this study was to develop rapid analytics (without manual grouping or repeated regression analysis) to visualize the key trends and relationships between aphid population and leaf damage scores with respect to perturbation parameters (genotype x year x environment).

## Materials and Methods

Field experimental design and juice and biomass characterization procedures were described in detail previously^22,37,38^, and are summarized in Section I of Supporting Information.

### Field scoring of sugarcane aphid population and leaf damage

In both 2015 and 2016, aphid damage was rated every other week (starting July 13, 2015 and July 1, 2016) using a visual rating scale similar to that described by Armstrong *et al*.^13^, where 1 = no damage; 1.5 = a small amount of honeydew on lower leaves; 2 = significant amount of honeydew on lower leaves with some leaf discoloration; 2.5 = up to 40% of lower leaves discolored and heavy honeydew present; 3 = up to 50% leaves discolored and sooty mold may be present; 3.5 = up to 60% leaves discolored and sooty mold present on leaves and ground; 4 = up to 75% leaves discolored, heavy sooty mold present, and some flowering may be aborted; 4.5 = aphid damage up to the flag leaf and most flowering aborted; 5 = plants dead, or nearly dead, from aphid damage. Aphid population was rated every other week (starting July 27, 2015 and June 17, 2016), using a semi-logarithmic scale: n = 0, ≤25, ≤50, ≤100, ≤500, ≤1000, and ≥5000. It must be noted that insecticide application is recommended at 50–125 aphids per leaf, or at 20–30% infestation with substantial honeydew^10^. Once the density exceeds 500 aphids per leaf, the exponential growth of aphids makes insecticide application impractical^10^.

Prior to analysis, the data were transformed using the formula y = log(n + 1), where n is the highest number in the class, or n = 5000 for the highest class. In 2016, population was estimated separately for the leaf just below the flag leaf (top), the lowest green leaf (bottom), and the average of these for approximately five plants per plot. Only the average was estimated in 2015. Early planted plots have fewer data points because they were harvested earlier (e.g., the April planting in 2015 was completely harvested by Aug. 17).

### PLS modeling of sugarcane aphid population and leaf damage

The PLS model was built using MATLAB version 8.6.0.267246 (R2015b; Mathworks, Natick, MA) with PLS toolbox version 8.6.2 (Eigenvector Research, Manson, WA). The VIP and regression vector outputs of PLS^34^ were used to explore the relationships between sugarcane aphid population and damage (independent variables, X) and chemical parameters^22,37,38^ (dependent variables, y). Independent variables (X) were biweekly aphid population or damage scores as a function of sampling date for a given planting month (April, May, or June in 2015, and May in 2016). The dependent variables (y) were previously reported chemical properties of juice and biomass: pH, electric conductivity (EC), and Brix; concentrations of glucose, fructose, sucrose, total sugar, and trans-aconitic acid; UV/visible absorbance of juice and bagasse; electrochemical parameters; and contributions of fingerprints determined by fluorescence excitation and emission (EEM) with parallel factor analysis (PARAFAC)^22,37,38^. Both variables were subjected to PLS without pre-processing, and the dataset was split into calibration and validation sets while keeping the replicates together. The built calibration model was cross-validated by the Venetian blinds with 8 data splits. Chemical parameters to be used as the dependent variable, and the number of latent variables were selected based on the root mean square errors of calibration (RMSEC), cross validation (RMSECV), and prediction (RMSEP), calibration and cross validation bias, r^2^ of calibration and cross validation, and by residual analysis. For the selected dependent variable and the number of latent variables, a VIP score above 1 was set as the threshold for interpretation. The regression vector was used to assign a positive or negative direction for the sampling dates exceeding the VIP threshold. Selectivity ratio was used to confirm the VIP threshold.

### ANOVA and correlation map

To confirm the interpretation of PLS, the major effects were examined by one-way or factorial (for cultivar x year interactions) ANOVA using Statistica version 12 (Statsoft, Tulsa, OK) at a significance level of p < 0.05. Type VI sums of squares was used to test the effective hypothesis for unbalanced observations. If significant differences existed, post hoc comparison by Tukey’s HSD test was performed with cultivar or year as the categorical factor. Pearson’s correlation (p < 0.05) was examined by Statistica, and a correlation matrix pseudocolor map was constructed using MATLAB with PLS toolbox. Variables were reordered by the similarity in Pearson’s r values using a modified k-nearest neighbor algorithm.

### Solid-phase EEM/PARAFAC of leaves

Reflective fluorescence EEM of leaf powder (<2 mm particle size) for April, May, and June plantings of 2015 were collected using the previously described method^22^ with the following modifications. The spectrofluorometer (F-7000; Hitachi, San Jose, CA) was set to 250–500 nm excitation and 280–730 nm emission wavelengths in 3 nm intervals; 5 nm EEM slits; auto response time; and 2400 nm min^−1^ scan rate. The quartz window on the metal sample cell (without sample) was used as the blank and its value was subtracted from each sample EEM. Additional regions dominated by Rayleigh and Raman peaks and the region without fluorescence were removed. Preprocessed spectra were used to model PARAFAC^39^ with non-negativity constraint using MATLAB with PLS toolbox. Of 197 spectra collected with 5 nm EEM slit widths, 7 exceeded the maximum intensity, and those samples were removed from PARAFAC. On the basis of residual/leverage analyses of 2–7 component models, a three-component model (72 core consistency) was selected for interpretation.

## Results and Discussion

### PLS examination of aphid population and leaf damage

**Figure 1:**
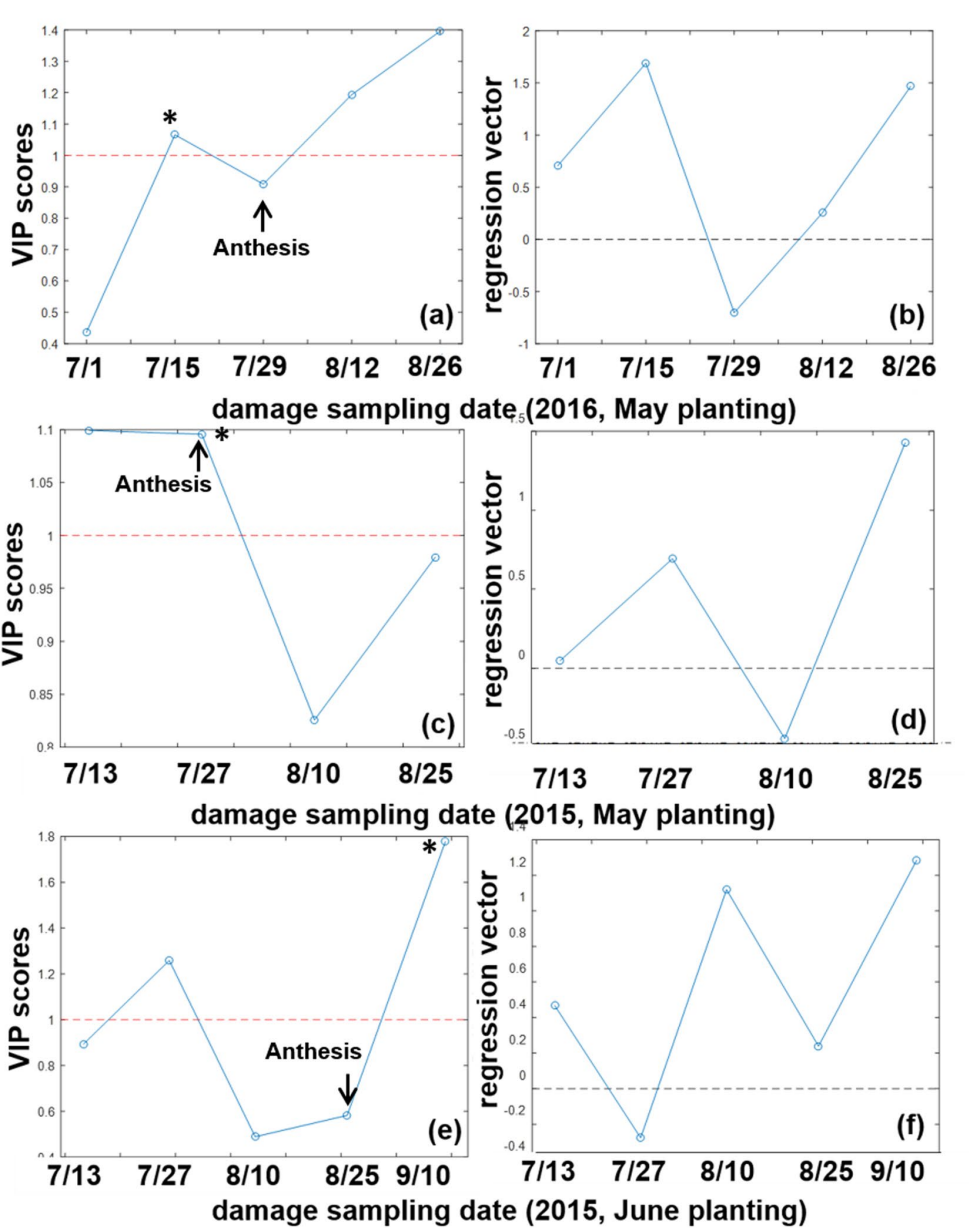
VIP scores and regression vectors for the damage ratings of (**a**,**b**) 2016 May planting, (**c**,**d**) 2015 May planting, and (**e**,**f**) 2015 June planting. Horizontal lines indicate the threshold for VIP (>1) and 0 for regression vector directionality. Arrows indicate approximate anthesis days. Asterisks indicate the first day fulfilling VIP > 1 and positive regression vector. Lines connecting data points are for visual aid, and do not represent model fits.

Sweet sorghum genotypes (cultivars) employed in this study are listed in Table S1, Supporting Information. Representative raw data for leaf damage and aphid population scores (Section II, Supporting Information) indicated large replication errors. Therefore, PLS was utilized as a rapid analytic method to (1) interpret the time trends (biweekly scores) of aphid population and damage and (2) examine the relationships between the aphid population and plant host damage. The goal of developing a new PLS analytic was to rapidly detect the meaningful data range and its directionality^29^. It must be noted that when PLS was employed for this purpose (as opposed to calibration and prediction), previous studies^33,34,40^ did not report the number of latent variables, r^2^, or scatter plots. In Fig. 1, PLS was built for a given planting month of each year. Regardless of the dependent variable (y), similar time trends in VIP, regression vector, and selectivity ratio were observed for a given independent variable (X), i.e., aphid population or damage score as a function of biweekly sampling dates from planting to harvest.

Figure 1 presents VIP scores (a) and regression vector (b) for the damage rating of the May 2016 planting sampled between July 1 and Aug. 26, 2016. Each plot was harvested at the hard dough stage between Aug. 15 and Sept. 27, 2016. Representative trends of the damage scores (raw data before PLS), scatter plots, latent variable, and selectivity ratio are presented in Fig. S1 of Supporting Information. Of all the chemical parameters^22,37,38^ considered, EC with 3 latent variables was selected because it had the highest r^2^ of calibration and cross validation. As shown in Fig. 1a, July 15, Aug. 12, and Aug. 26, 2016 exceeded the VIP threshold of 1 with corresponding positive regression vectors in Fig. 1b. These trends suggest increasing damage (positive “slope”) at a later growth stage (3 months after planting). Figure 1c,d show analogous PLS models for the May planting of the preceding year (2015; pH as y-variable, Fig. S2). In contrast to 2016, earlier growth stage (July 13, 2015 with zero regression vector, and July 27, 2015 in the positive direction) exceeded the VIP threshold. Figure 1e,f provide analogous plots for a different planting date (June) of 2015 sampled between July 13 and Sept. 10, 2015 (EC as the dependent variable, Fig. S3). The April planting of 2015 did not have enough data points to perform PLS. Similar to the May planting of 2016 (Fig. 1a,b), significant (>1) VIP scores were observed in both early (July 27, 2015; negative regression vector in Fig. 1f) and late (Sept. 10, 2015; positive regression vector) growth stages in Fig. 1e.

**Figure 2:**
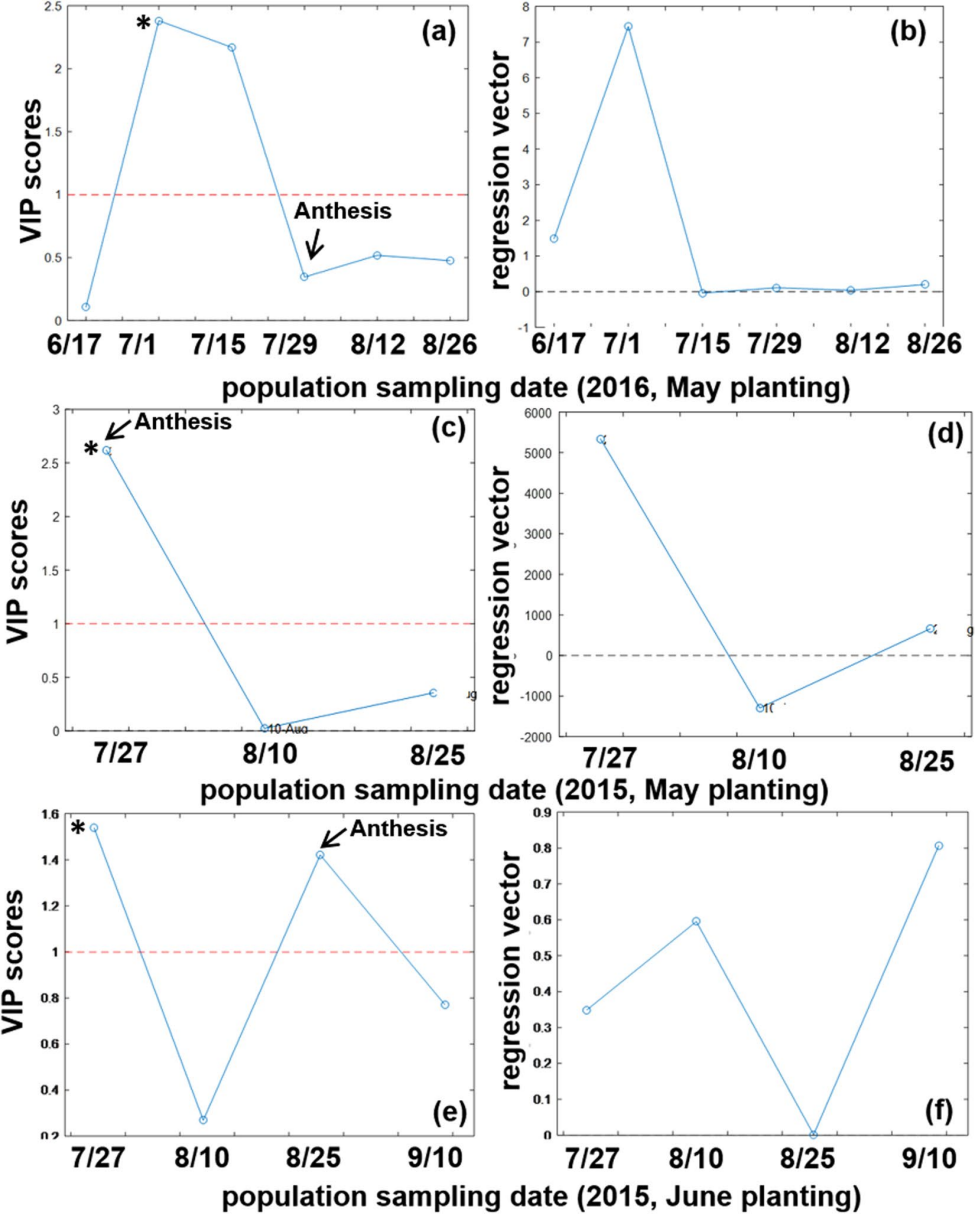
VIP scores and regression vectors for the aphid population time courses for (**a**,**b**) (n + 1) in 2016 May planting, (**c**,**d**) log (n + 1) in 2015 May planting, and (**e**,**f**) (n + 1) in 2015 June planting.

Figure 2 presents PLS results for population time courses to provide a comparison to damage in Fig. 1. Figure 2 is arranged in the same order as Fig. 1: (a-b) 2016 May planting, (c-d) 2015 May planting, and (e-f) 2015 June planting. The left panels show VIP scores, and the right panels show the regression vector in both Figs 1 and 2. In Fig. 2, population (n + 1) without log transformation was used for 2016 May (a-b) and 2015 June (e-f), while log (n + 1) was used for 2015 May (c-d). The dependent variables for PLS in Fig. 2 were reduction potential (E_h_) (a-b), bagasse PARAFAC factor 2 contribution (c-d), and bagasse PARAFAC %1 contribution (e-f); additional PLS statistics are provided in Figures S4–S6 of Supporting Information. In contrast to damage (Fig. 1), VIP scores of population exceeded the threshold at early growth stage (in July, Fig. 2a,c), except for Aug. 25, 2015 in Fig. 2e having zero regression vector. Highly positive regression vectors (Fig. 2b,d) for early growth stage indicate that population increase preceded damage occurring at a later growth stage. In conclusion, regardless of planting month or year, population increase (positive regression vector having VIP score above 1, marked * in Figs 1 and 2) occurred on or before the day fulfilling both criteria for damage: July 1, 2016 population followed by July 15, 2016 damage for the 2016 May planting; July 27, 2015 population and damage for the 2015 May planting; and July 27, 2015 population and Sept. 10, 2015 damage for the 2015 June planting. Approximate anthesis days (70 ± 8 days after planting in 2015; 68 ± 13 days in 2016) are marked as arrows in Figs 1 and 2. Significant damage (fulfilling both VIP above 1 and positive regression vector, marked * in Figs 1 and 2) occurred before flowering in 2016 (May planting, Fig. 1a), and on or after flowering in 2015 (May and June plantings, Fig. 1c,e). Sucrose and biomass reportedly accumulate in stems after floral induction^41^, when growth of stems and leaves is complete, and sucrose-hydrolyzing enzymatic activity decreases. Aphid damage before flowering in 2016 could have contributed to the lower sugar production observed^42^ in 2016 than in 2015^22^.

**Figure 3:**
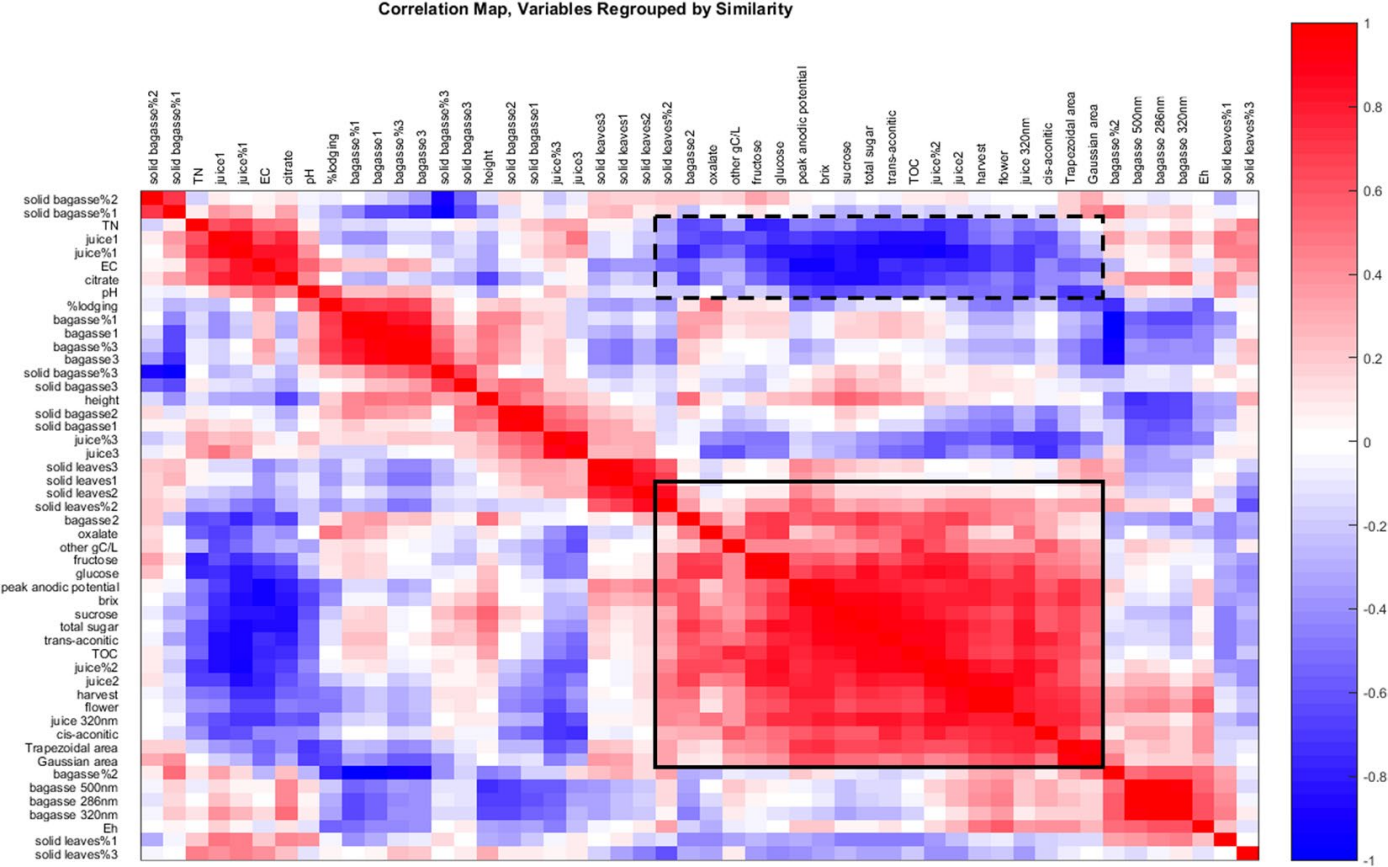
Correlation map (Pearson’s p < 0.05) for all investigated parameters in April, May, and June plantings of 2015. Darker red indicates strongly positive correlation (towards r = 1), while darker blue indicates strongly negative correlation (towards r = −1). The following ranges (represented by white region) are not significant at p < 0.05: r = 1 and −0.3 to 0.3.

To further explore the impact on chemical composition, Fig. 3 provides a correlation (Pearson’s) map for all chemical and growth parameters investigated on 2015 April, May, and June plantings. Darker colors indicate greater linear relationships in positive (red) and negative (blue) directions; r = 1 and −0.3 to 0.3 (white regions) are not significant at p < 0.05. The solid black rectangle in Fig. 3 highlights a region having the largest number of positively correlated variables: electrochemical parameters (Gaussian and trapezoidal areas and peak anodic potential, E_pa_), sugars (sucrose, glucose, fructose, Brix, total sugar), total organic carbon (TOC), carboxylates (trans- and cis-aconitic and oxalic acids), aromatic juice and bagasse EEM/PARAFAC fingerprints, growth parameters (days to flowering and harvest) and UV absorbance of juice (320 nm). Those parameters are negatively correlated to pH, EC, citric acid, total nitrogen (TN), and mid-aromaticity juice PARAFAC fingerprints (blue region surrounded by a dashed black rectangle). Therefore, chemical parameters attributable to organic carbon (sugars, primary carboxylates, and polyphenols) positively correlated with one another; whereas those parameters were negatively correlated with inorganic parameters such as pH, EC and TN.

Of all available chemical parameters^22,37,38^ tested as the dependent variable of PLS (Figs 1 and 2), inorganic parameters (EC and pH) best modeled the observed leaf damage, while parameters attributable to the redox reactivity (E_h_ and aromatic EEM/PARAFAC fingerprints) best modeled the aphid population. Therefore, the leaf damage trend was best modeled by alkali and alkaline earth metals accumulated at the expense of organic carbon products. On the other hand, aphid population was best modeled by the redox active components of sweet sorghum. Our previous study showed as much as 7-fold higher electron donating capacity of No. 5 Gambela (Table S1), a genotype used as the aphid-resistant control^22^. Electroactive polyphenolic structures could form upon aphid infestation, and subsequent damage could involve the accumulation of inorganic products in sweet sorghum juice. However, pest-host interactions involve predators in the higher ecological food chain, in addition to phytochemistry (defense phytochemicals)^43,44^. For example, some aphids accumulate glucosinolates to provide defense against predators, which causes a net cost to the host plant by promoting the survival of aphids^43,44^. Future research could explore the relationships between the chemical composition of sweet sorghum and resistance to aphids by utilizing emerging field-deployed hyperspectral or thermal imaging techniques.

### Confirmation of PLS interpretations

One-way ANOVA was used to confirm PLS-based data analytics (Figs 1 and 2) and to describe cultivar effects. Cultivar effects were examined for each population and damage rating, and replication was analyzed as a random effect. Table 1 presents biweekly population and damage scores showing significant (p < 0.05) cultivar effects for a given planting: April, May, or June for 2015, and May for 2016. In 2016, cultivar main effects on damage were observed near harvesting (Aug. 26, 2016 and Sept. 9, 2016) at the hard-dough maturity stage; however, no significant differences between cultivars were detected by Tukey’s HSD procedure. In contrast, cultivar main effects on 2016 aphid population were observed at early growth stage (June 17) for the top leaves (with and without log-transformation). At a later growth stage (Aug. 12), the hybrid N109A x Isidomba was infested with a significantly higher aphid population (333 mean count) than all other cultivars examined.

**Table 1:** Significant p-value (<0.05) for cultivar main effects (far right column) with post-hoc Tukey’s HSD test for each biweekly leaf damage and aphid population scoring date (from planting to harvest) for a given planting month (April, May, or June) of 2015 or 2016.

method	year	planting	scoring	harvest	n	mean	s.d.	min	max	cultivar Tukey p<0.05 (mean)	cultivar
*damage*	2015	4/22	7/13	7/23-8/13	69	3.2	0.4	2.0	3.8		0.006
(score 1-5)											
	2015	5/14	7/13	8/6-8/27	69	3.0	0.6	1.5	4.0	N109B (3.9), Chinese (3.8) > Isidomba (2)	0.035
	2015	5/14	8/10	8/6-8/27	69	2.6	0.6	1.0	3.5		0.018
	2015	5/14	8/25	8/6-8/27	69	2.8	0.4	1.5	3.5	all (except Atlas,N111AxAtlas,N109AxAtlas,N111AxDale,N111AxIsidomba) >Isidomba (1.8)	0.005
	2015	6/16	9/10	9/9-9/24	69	2.2	0.6	1.0	3.5	N111B, N109AxChinese, N109AxN98 > Isidomba (1.2)	0.016
	2016	5/17	8/26	8/15-9/27	72	2.5	0.9	1.0	4.0		0.022
	2016	5/17	9/9	8/15-9/27	42	2.4	0.9	1.0	3.5		0.041
*population*											
average	2015	6/16	9/10	9/9-9/24	69	18.4	19	1	101	N109AxIsidomba (mean=59.3)>Atlas,Dale,Isidomba,N110B,N111B, N109AxN98,N110AxDale,N110AxIsidomba,N110AxN98,N111AxN98	0.005
average-log	2015	6/16	9/10	9/9-9/24	69	0.8	0.8	0.0	2.0		0.012
top	2016	5/17	6/17	8/15-9/27	72	6	11	0	25		0.023
top-log	2016	5/17	6/17	8/15-9/27	72	0.3	0.6	0.0	1.4		0.023
top	2016	5/17	8/12	8/15-9/27	72	41	99	0	500	N109AxIsidomba (mean=333)>all others	0.006

Leaf damage was rated as 1 (no damage) to 5 (severe damage or dead plant), and aphid population was reported both without (n + 1) and with log transformation. Cultivar Isidomba was most resistant to damage in 2015. Cultivar N109A × Isidomba had the highest aphid population in both 2015 and 2016. All dates below are in month/day format.

For 2015, the cultivar Isidomba exhibited resistance to aphid damage (mean damage = 2), relative to the most susceptible genotypes: Chinese (mean = 3.83) and N109B (mean = 3.92) in early growth stage (July 13, 2015) of the May planting. Cultivar effects were observed again at later growth stage of the May planting (Aug. 10 and Aug. 25, 2015) when Isidomba (lowest damage, 1.83) again exhibited resistance or tolerance compared to all other genotypes except Atlas, N111A x Atlas, N109A x Atlas, N111A x Dale, and N111A x Isidomba. For the June planting of 2015, the lowest damage was observed again for Isidomba (1.12 mean damage) at late growth stage (Sept. 10), compared to the most susceptible genotypes: N111B, N109A x Chinese, and N109A x N98. In summary, Isidomba consistently showed resistance or tolerance to aphid damage in the later growth stage of 2015. However, the cultivar most resistant to damage (Isidomba) did not have the lowest aphid population in 2015 or 2016 (Table 1). Likewise, the cultivar with the highest aphid population (N109A x Isidomba in both 2015 and 2016) did not show the highest damage. In conclusion, ANOVA could not deduce clear relationships between aphid population and leaf damage.

Table 2 presents significant (p < 0.05) cultivar, year, and interaction effects on aphid population and leaf damage scored on approximately the same sampling days of 2015 and 2016 for the May plantings. No. 5 Gambela was planted in 2016 but not in 2015 and was removed from the factorial ANOVA analysis in Table 2. At the earliest growth stage (July 13, 2015 vs. July 15, 2016 for May plantings), the mean damage rating was lower in 2016 than 2015. On July 27, 2015 vs. July 29, 2016, both damage and population were lower in 2016. However, at the next set of sampling dates in August, population was greater in 2016, and cultivar N109B in 2016 had significantly higher aphid population count, compared to all other genotype x year pairs. At the latest growth stage (Aug. 25, 2015 vs. Aug. 26, 2016 for August-September harvesting), damage was lower, while population was higher in 2016, and N109B had significantly higher damage than Isidomba, as shown in Table 1. In conclusion, the examined sweet sorghum cultivars sustained less apparent damage in 2016 than 2015.

**Table 2:** Significant (p < 0.05) cultivar, year, and interaction main effects by factorial ANOVA and post-hoc Tukey’s HSD test for given leaf damage and aphid population scoring days of May plantings in 2015 and 2016.

scoring date	variable	n	mean	s.d.	min	max	non-zero	significant (<0.05) p value		
								cultivar	year	interaction
7/13/15, 7/15/16	damage^a^	138	3	1	1	4	138		<0.001 (↓)	
7/27/15, 7/29/16	damage	138	3	1	1	4	138		<0.001 (↓)	
	population (average)	138	26	52	1	501	138		<0.001 (↓)	
	population (average-log)	138	1	1	0	3	73		<0.001 (↓)	
8/10/15, 8/12/16	population (average)	138	29	71	1	501	138	0.043	<0.001 (↑)	0.044 (N109B in 2016>all others)
	population (average-log)	138	1	1	0	3	53		<0.001 (↑)	
8/25/15, 8/26/16	damage	138	3	1	1	4	138	0.012 (N109B>Isidomba)	0.024 (↓)	
	population (average)	138	27	42	1	301	138		<0.001 (↑)	
	population (average-log)	138	1	1	0	2	82		<0.001 (↑)	

Planting dates were May 14, 2015 and May 17, 2016. Harvest dates (hard-dough stage of maturity) were Aug 6–27, 2015, and Aug. 15–Sept. 27, 2016. Scoring dates below are listed in month/day/year format.

^a^Population data for this date were only available for 2016.

Collectively, one-way ANOVA confirmed the following conclusions drawn from PLS modeling. First, higher damage was observed at later growth stage (Table 1 and Fig. 1a,b). Second, greater damage at earlier growth stage in 2015 (Fig. 1c,d) than 2016 (Fig. 1a,b) likely led to higher overall damage ratings observed in 2015 than 2016 (Table 2). Finally, later growth stage (Sept. 10, 2015 in Fig. 1e,f showing positive regression vector indicating increasing damage) showed significant cultivar effects, where Isidomba was most resistant to damage (Table 1).

### Fluorescence EEM/PARAFAC of leaves

**Figure 4:**
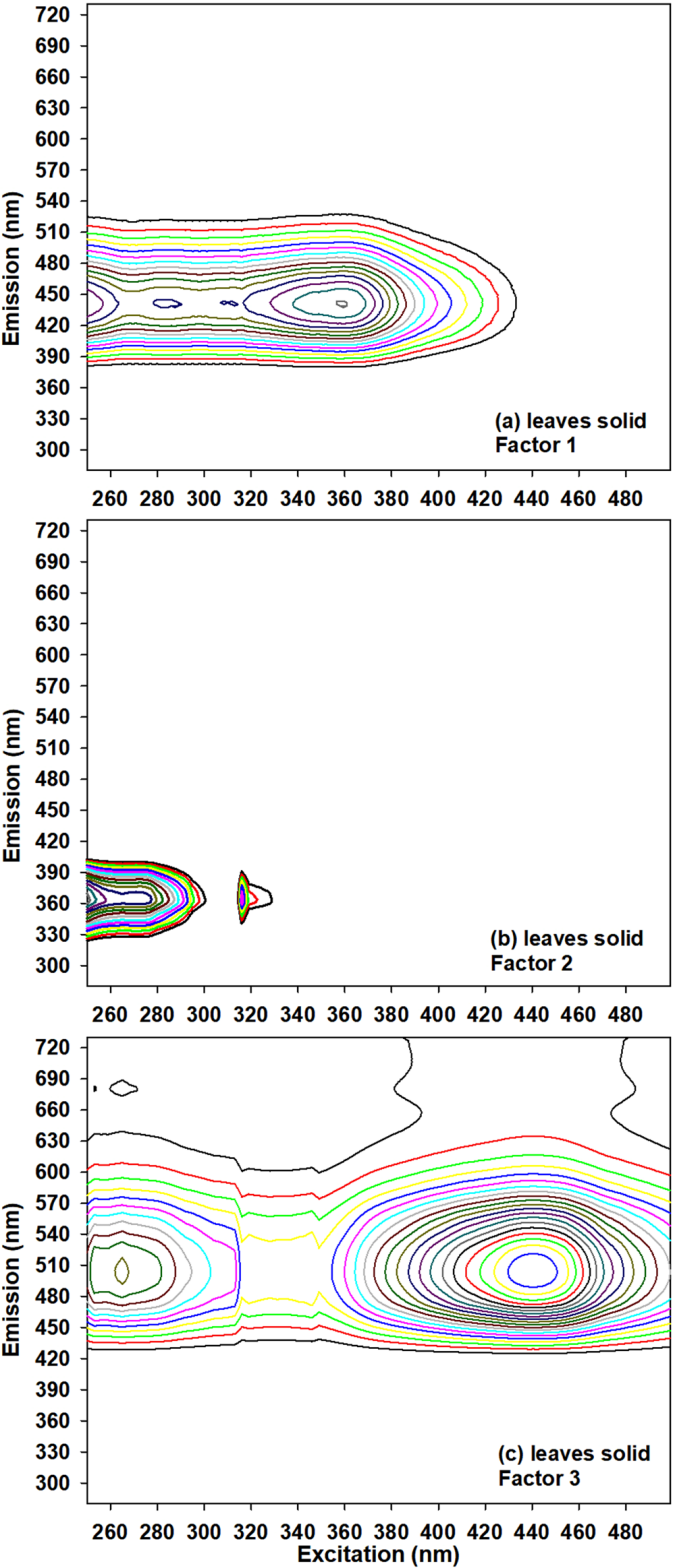
Solid-phase EEM/PARAFAC fingerprints obtained from dry leaf powder (<2 mm particle size) of 2015 April, May, and June plantings.

Colonization and stylet penetration by aphids occur on leaves^20^, resulting in visible symptoms of damage, which were assessed by assigning visual damage ratings (Fig. 1). Therefore, dry leaves (April, May, and June plantings of 2015) were analyzed by *in situ* solid-state reflective fluorescence EEM/PARAFAC without chemical extraction. Figure 4 presents fingerprint structures having most aliphatic (Fig. 4b), mid-range (Fig. 4a), and most aromatic/conjugated structures (Fig. 4c). As described in detail in Section V of Supporting Information, cultivar effects (by ANOVA) for fluorophores in leaves (Fig. 4) did not follow that of aphid population or leaf damage scores (Table 1). However, correlation (Pearson’s p < 0.05, Fig. 3) with reported growth factors^22^ could be used to predict agronomic parameters from chemical fingerprints, and vice versa. Mature plant height positively correlated (r = 0.17) with the aromatic fluorophores in leaves (absolute contributions of factors 1 and 3 in Fig. 4). Lodging percentage correlated (r = 0.16) with % contribution (normalized to the contributions of all 3 fingerprints in Fig. 4) of aliphatic structure (Fig. 4b). Furthermore, greater days to anthesis (harvest occurred ≈ 30 d after flowering) and taller plant height paralleled greater accumulation of organic carbon products, while lodging percentage had an opposite effect (Fig. 3).

In conclusion, PLS modeled aphid population increase preceding observed leaf damage at a later growth stage. Overall, sweet sorghum cultivars sustained less damage in 2016 than 2015. This yearly trend in damage coincided with higher concentrations of trans-aconitic acid and polyphenolic secondary products in 2016 than 2015, possibly at the expense of primary sugar (sucrose, glucose, and fructose) production^22^. Those secondary products could serve as anti-feedant defensive phytochemicals against aphids^21,25^. Greater damage at earlier growth stage in 2015 than 2016 likely further contributed to the overall higher damage rating in 2015 than 2016. Results indicated the interplay of sorghum’s growth stage, genotypic resistance, and planting date on observable damage by sugarcane aphids.

### Supporting Information Available

Methods; representative trends of the damage scores (raw data before PLS), scatter plots, latent variable trends, and selectivity ratio; representative trends of population scores (raw data before PLS), scatter plots, latent variable trends, and selectivity ratio; and factorial ANOVA (pedigree, planting) results for EEM/PARAFAC fingerprints of leaves (Fig. 3). This materials is available free of charge via the Internet.

### Disclaimer

Mention of trade names or commercial products in this publication is solely for the purpose of providing specific information and does not imply recommendation or endorsement by the U.S. Department of Agriculture. USDA is an equal opportunity provider and employer.

## Electronic supplementary material

SUPPLEMENTARY INFORMATION
