# Supplementary material for: Rapid Data Analytics to Relate Sugarcane Aphid [(Melanaphis sacchari (Zehntner)] Population and Damage on Sorghum (Sorghum bicolor (L.) Moench)
Source: Sci Rep. 2019 Jan 23;9:370. doi: 10.1038/s41598-018-36815-0 (PMC6344576; doi:10.1038/s41598-018-36815-0)
Supplement: Supplementary file 1 — SUPPLEMENTARY INFORMATION [file 41598_2018_36815_MOESM1_ESM.doc]

Supporting Information for

Rapid Data Analytics to Relate Sugarcane Aphid [(*Melanaphis sacchari* (Zehntner)] Population and Damage on Sorghum (*Sorghum bicolor* (L.) Moench)

Minori Uchimiya^*,a^ and Joseph E. Knoll^b^

*^a^USDA-ARS Southern Regional Research Center, 1100 Robert E. Lee Boulevard, New Orleans, LA 70124*

*^b^USDA-ARS Crop Genetics and Breeding Research Unit, 115 Coastal Way, Tifton, GA 31793*

*Corresponding author fax: (504) 286-4367, phone: (504) 286-4356, email: sophie.uchimiya@ars.usda.gov (M. Uchimiya)

Number of pages: 15

Number of tables: 1

Number of figures: 6

**I. Methods**

**Field experiment.** A two-year field experiment was conducted near Tifton, GA (31° 29’ N, 83° 31’ W) on a Tifton loamy sand (fine-loamy, kaolinitic, thermic Plinthic Kandiudults) with the following modifications to the experimental design described in detail previously (Knoll and Anderson, 2016; Uchimiya et al.). Fifteen hybrids were generated by a paper bag pollination using male-sterile female seed parents (N109A, N110A, and N111A) (Gorz et al., 1990), each crossed to five inbred lines (Atlas, Chinese, Dale (Broadhead and Coleman, 1973), Isidomba, and N98) (Gorz et al., 1990) as males. The inbred parents (male-fertile (B-line) versions of the female lines) were also planted, for a total of 23 entries (Table S1) planted in a split-plot design with three replications with planting dates (April, May, or June) as the main plot factor and cultivars as the subplot factor in 2015. In 2016, an additional cultivar (No.5 Gambela, Table S1) was planted to test for its resistance to sugarcane aphid pest colonization and injury. Planting dates were April 22, May 14, and June 16 in 2015, and May 17 in 2016. Each subplot was 6 m long, and rows were 0.9 m apart. Subplots consisted of two rows in 2015, but were reduced to one row in 2016 due to a limited supply of seed. Prior to planting, granular PKN (10-10-10) fertilizer was applied at a rate of 560 kg ha^-1^. At ≈30 d after planting, additional N was applied by a side-dress liquid application (112 kg ha^-1^ N). Weeds were controlled by the herbicides pendimethalin (N-(1-ethylpropyl)-3,4-dimethyl-2,6-dinitrobenzenamine), atrazine (1-chloro-3-ethylamino-5-isopropylamino-2,4,6-triazine), and bentazon (3-isopropyl-1H-2,1,3-benzothiadiazin-4(3H)-one 2,2-dioxide). Irrigation was applied only as needed to speed germination, and no insecticides were used.

Harvest dates were set to the hard-dough stage of maturity (when Brix typically peaks), and occurred on August 6, 13, 20, and 27 in 2015; and August 15, 23, and 30, and September 6, 13, 20, and 27 in 2016. Three representative stalks were harvested from each subplot, panicles and leaves were removed, and juice was extracted from the stems by passing twice through a portable three-roller mill (Sor-Cane Porta-Press, McClune, Reynolds, GA). Juice samples were immediately frozen after measuring the soluble solids concentration (Brix) using a digital refractometer (Refracto 30GS, Mettler-Toledo, Columbus, OH) (Knoll and Anderson, 2016). The bagasse portion was dried at 60 °C until the weight stabilized, ground in a Wiley mill (Thomas Scientific, Swedesboro, NJ), and sieved (<2 mm).

**Juice and bagasse characterization.** Characterization methods and results for 2015 samples have been reported (Uchimiya et al.; Uchimiya et al.) and analogous methods were used to characterize 2016 samples (corresponding to planting 2, i.e., May planting). Briefly, distilled, deionized water (DDW) with a resistivity of 18 MΩ cm (APS Water Services, Van Nuys, CA) was used in all laboratory procedures. All chemical reagents were obtained from Sigma-Aldrich (Milwaukee, WI) with the highest purity available. Because sweet sorghum juice is unstable at room temperature (Wu et al., 2010), samples were defrosted in a refrigerator, and were immediately diluted (large-orifice pipette tips, Fisher, Hampton, NH) by DDW, filtered (0.45 μm PVDF, Fisher), and analyzed. Sucrose, glucose, fructose, and citric, oxalic, trans- and cis-aconitic acid concentrations were quantified using an HPLC system with refractive index and diode array detectors (Agilent Technologies, Santa Clara, CA) and a Hi-Plex H column (Fisher) with 5 mM sulfuric acid mobile phase at 0.6 mL min^-1^ flow rate and 20 μL injection volume.

Electric conductivity (EC), pH, and reduction potential (E_h_ in mV) of juice samples were determined without dilution using YSI 3200 conductivity meter (YSI, Yellow Springs, OH) for EC; ORION ROSS Sure-Flow Glass Combination pH Electrode for pH; and an epoxy sure-flow combination redox/ORP electrode (9678BNWP, Fisher) for E_h_. Sartorius Professional PP-15 meter (Sartorius, Bohemia, NY) was used to record the electrode response for both pH and E_h_. Total organic carbon (TOC in ppm C) and total nitrogen (TN in ppm N) were analyzed using a Torch combustion TOC/TN analyzer (Teledyne Tekmar, Mason, OH). The TOC values were converted to “other gC/L” by subtracting the portion of TOC originating from sugars (sucrose, glucose, and fructose) (Uchimiya et al.).

Cyclic voltammograms of diluted (2-fold by DDW) juice samples (set to 0.1 M KCl and 40 mM phosphate buffer at pH 5) were obtained using a WaveNow Potentiostat (Pine Research Instrumentation, Durham, NC) with a disposable screen-printed carbon electrode (SPE, 2 mm with Ag/AgCl reference electrode). For each juice sample, a new SPE was sacrificed (Ugliano, 2016) to first collect background CV (0.1 M KCl and 40 mM phosphate buffer at pH 5) by exposing the solution to -0.5 V for 60 s, and subsequently increasing to 1.2 V, and then decreasing to -0.5 V at 100 mV s^-1^ sweep rate in both directions. Immediately after collecting the background CV, the CV of the juice sample was obtained by the same procedures. Each raw CV (in current (A) vs. potential (V)) was processed using OriginPro 2016 (OriginLab, Northampton, MA) by (1) background subtraction, (2) Savitzky-Golay smoothing of first derivative, and (3) trapezoidal integration at 0.5-1.0 V range. The derivative CV of juice was additionally processed by the Gaussian integration (OriginPro 2016) to obtain E_pa_ and corresponding peak area. Gaussian fitting of peaks employed the asymmetric least square smoothing of the baseline, peak selection by the second derivative, and error analyses by reduced Chi-square and residual plots.

Bagasse powder (<2 mm) was extracted by methanol (0.4 g to 20 mL) by 24 h end-over-end rotation (70 rpm) at 25 °C, and then filtered (0.45 μm PTFE, Fisher). UV/visible spectra (HP8452A, Hewlett-Packard, Palo Alto, CA) of juice (20-fold diluted) and bagasse (10-fold diluted) were obtained after blank subtractions with DDW (juice) or methanol (bagasse).

**Table S1.** Inbred and hybrid sweet sorghum varieties investigated in this study. Entries are color-coded to correspond to the figures. PI number was obtained from the National Plant Germplasm System (NPGS) accession identifier (https://www.ars-grin.gov). Table was modified from (Uchimiya et al., 2017b).

| Entry | Group membership | PI number | Reference |
| --- | --- | --- | --- |
| Atlas | Inbred male, sweet/forage cultivar | PI 641807 | (Knoll and Anderson, 2016) |
| Chinese | Inbred male, landrace | - | (Knoll and Anderson, 2016) |
| Dale | Inbred male, improved sweet cultivar | PI 651495 | (Broadhead and Coleman, 1973) |
| Isidomba | Inbred male, landrace | PI 144331 | (Knoll and Anderson, 2016) |
| N98 | Inbred male, improved breeding line | PI 535783 | (Gorz et al., 1990) |
| N109B | Inbred female, 3-dwarf seed parent | PI 535794 | (Gorz et al., 1990) |
| N110B | Inbred female, 2-dwarf seed parent | PI 535795 | (Gorz et al., 1990) |
| N111B | Inbred female, 2-dwarf seed parent | PI 535796 | (Gorz et al., 1990) |
| N109A x Atlas | Hybrid | - | (Knoll and Anderson, 2016) |
| N109A x Chinese | Hybrid | - | (Knoll and Anderson, 2016) |
| N109A x Dale | Hybrid | - | - |
| N109A x Isidomba | Hybrid | - | (Knoll and Anderson, 2016) |
| N109A x N98 | Hybrid | - | (Knoll and Anderson, 2016) |
| N110A x Atlas | Hybrid | - | (Knoll and Anderson, 2016) |
| N110A x Chinese | Hybrid | - | (Knoll and Anderson, 2016) |
| N110A x Dale | Hybrid | - | - |
| N110A x Isidomba | Hybrid | - | (Knoll and Anderson, 2016) |
| N110A x N98 | Hybrid | - | (Knoll and Anderson, 2016) |
| N111A x Atlas | Hybrid | - | (Knoll and Anderson, 2016) |
| N111A x Chinese | Hybrid | - | (Knoll and Anderson, 2016) |
| N111A x Dale | Hybrid | - | - |
| N111A x Isidomba | Hybrid | - | (Knoll and Anderson, 2016) |
| N111A x N98 | Hybrid | - | (Knoll and Anderson, 2016) |
| No.5 Gambela | Landrace zerazera | PI 257599 | (Rosenow et al., 1997; Wang et al., 2009) |

**II. Representative raw damage and population ratings for different genotypes in 2016**

**III. Representative trends of the damage scores (raw data before PLS), scatter plots, latent variable trends, and selectivity ratio**


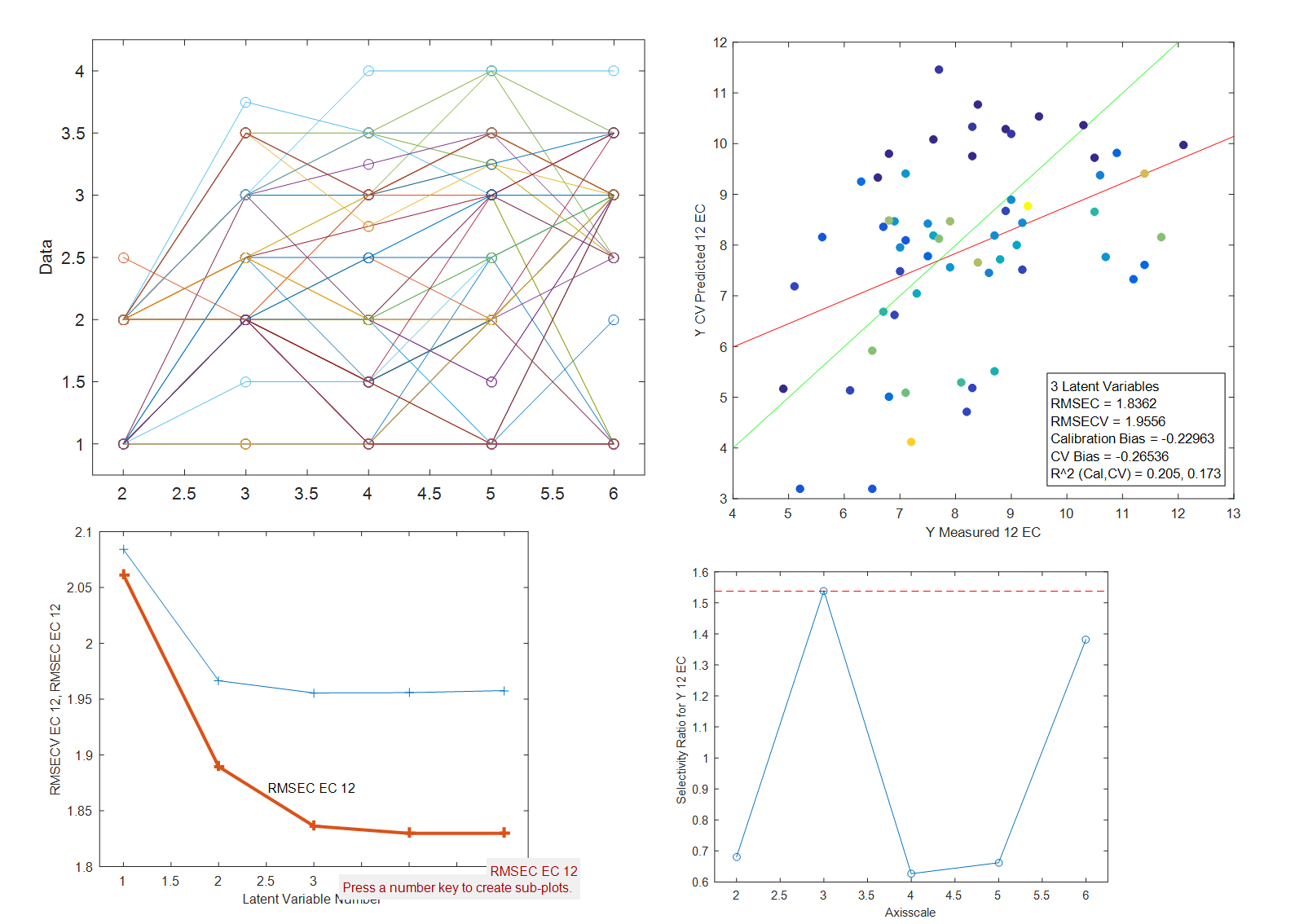


**Figure S1.** Damage for 2016 May planting (planting 2). X-axis of representative raw data (top left) and selectivity ratio (bottom right) are the sampling dates corresponding to: 2=7/1, 3=7/15, 4=7/29, 5=8/12, and 6=8/26. Lines are the PLS model fit (red) and 1:1 (green).


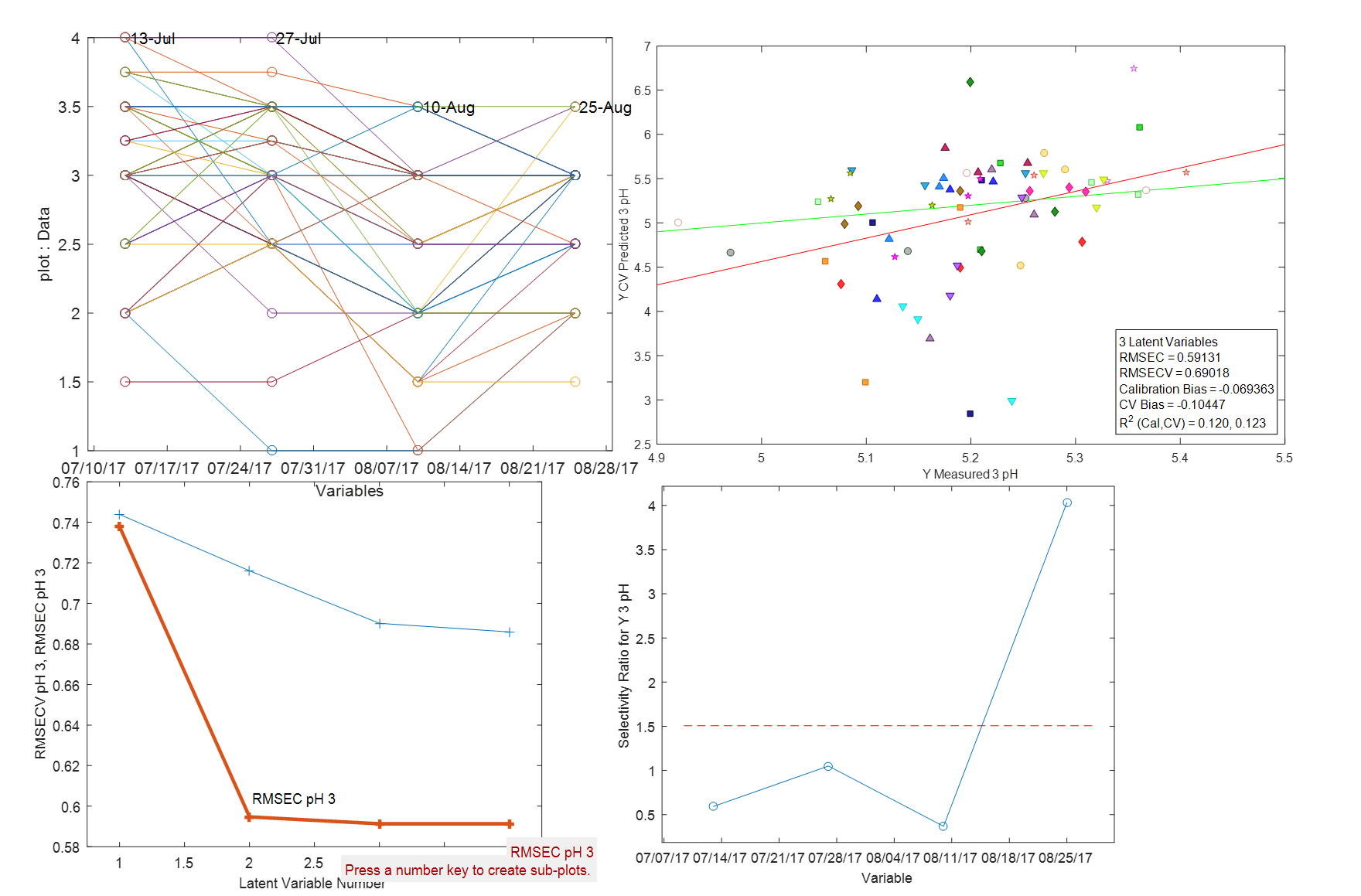


**Figure S2.** Damage PLS results for 2015 May planting (planting 2).


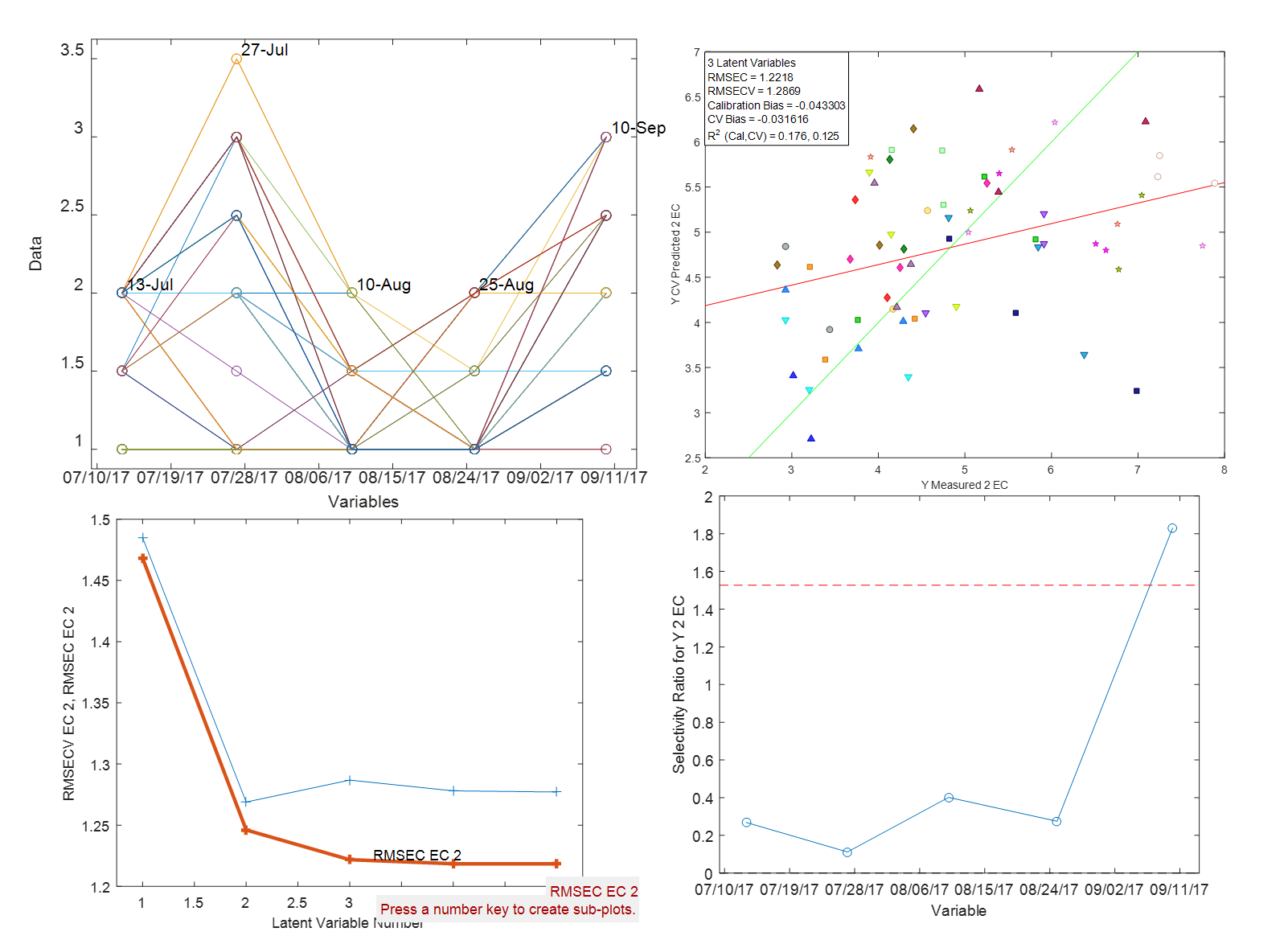


**Figure S3.** Damage PLS results for 2015 June planting (planting 3). Different colored symbols in the scatter plot (top right) are different cultivar classes.

**IV. Representative trends of population scores (raw data before PLS), scatter plots, latent variable trends, and selectivity ratio**


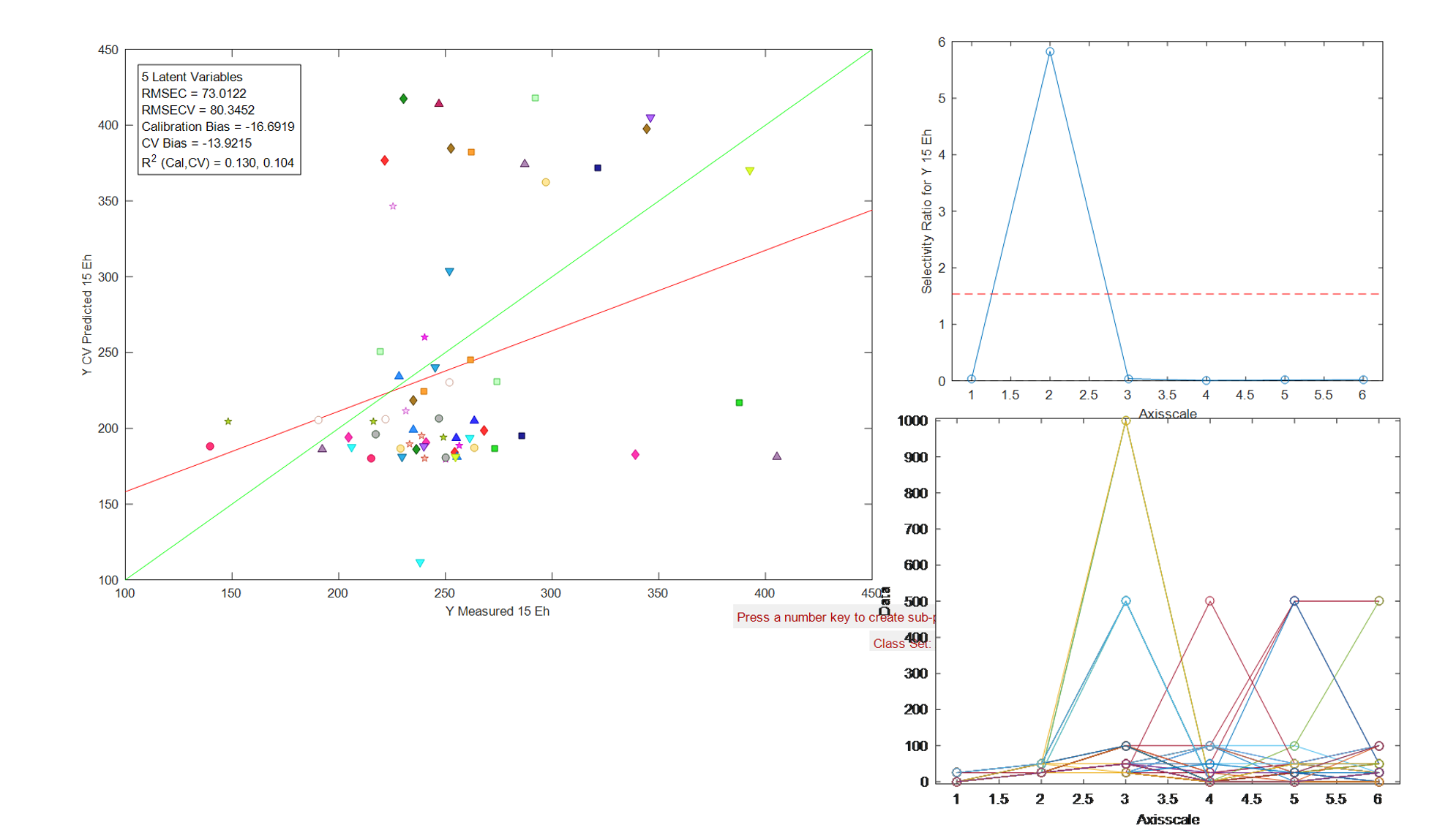


**Figure S4.** Population (n+1, without log transformation) for 2016 May planting (planting 2).


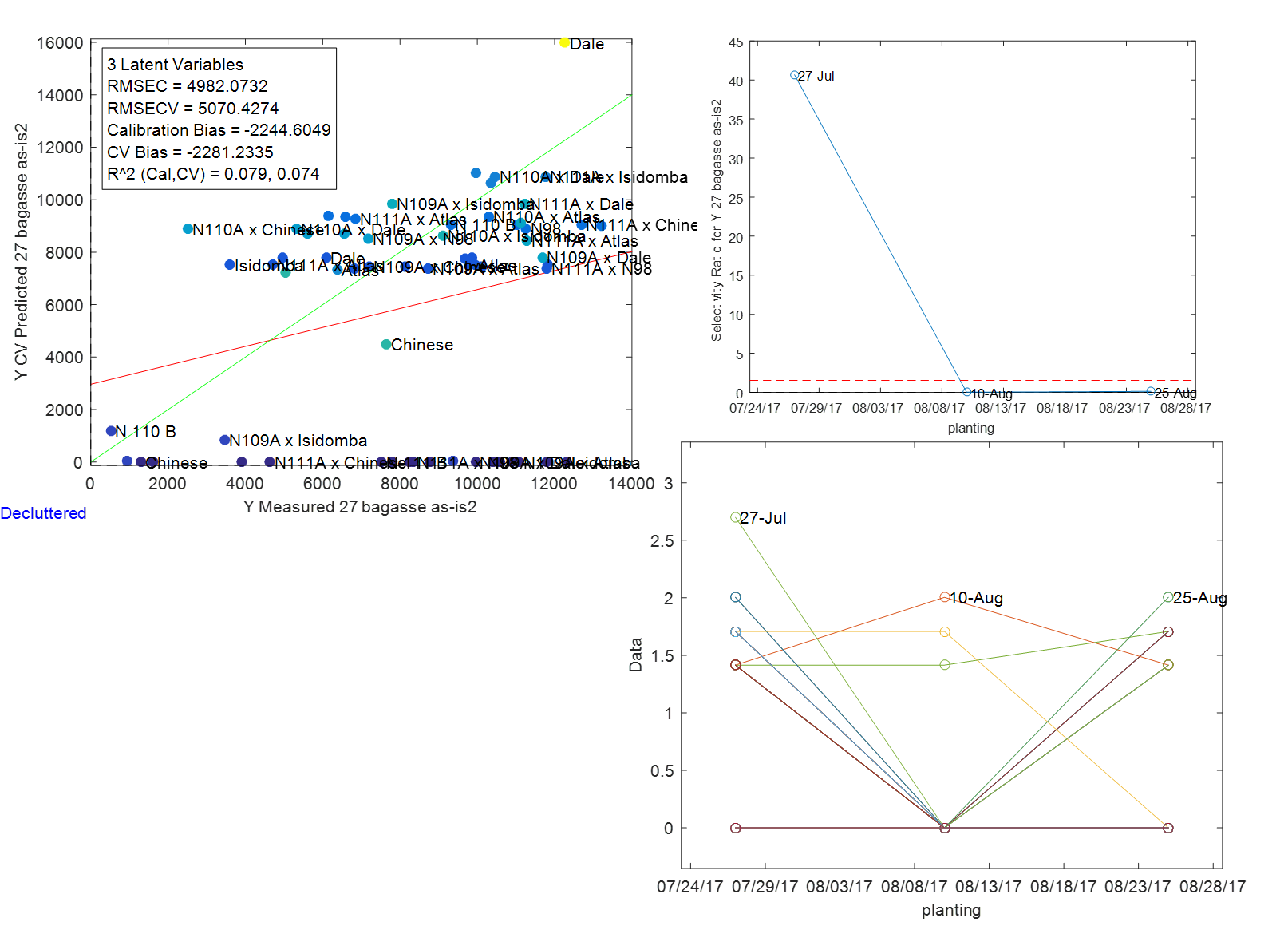


**Figure S5.** Population (n+1, without log transformation) for 2015 May planting (planting 2).


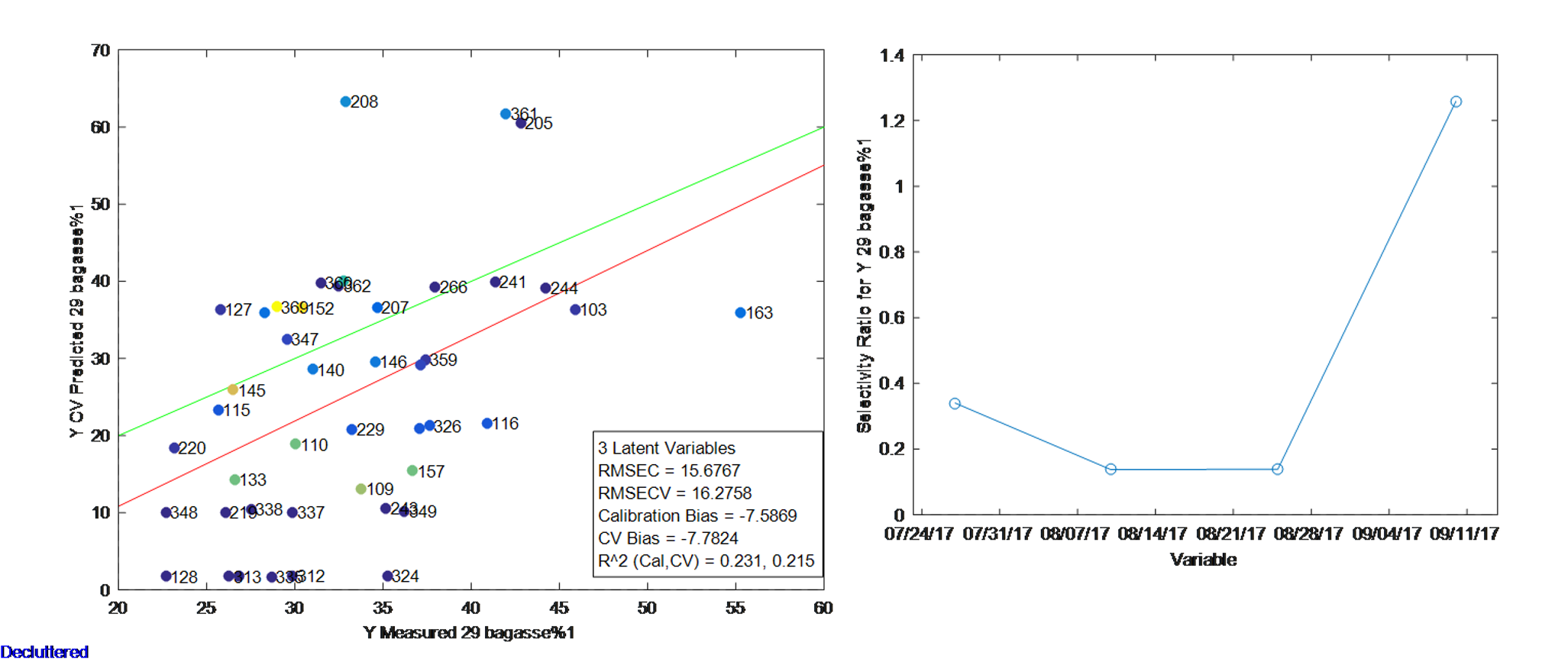


**Figure S6.** Population (n+1, without log transformation) for 2015 June planting (planting 3).

**V. Factorial ANOVA (pedigree, planting) results for EEM/PARAFAC fingerprints of leaves (Figure 3)**

Cultivar effects for sugarcane aphid population and damage (Table 1) did not show clear relationships with the cultivar effects for fluorescence chemical fingerprints (Figure 3) in the leaves of sweet sorghum:

Absolute factor 1 pedigree p=0.00154; no Tukey effects.

Absolute factor 1 planting p=0.00624; planting 1 > planting 3.

Absolute factor 2 pedigree p=0.02867; no Tukey.

%factor 1 planting p=0.01383; planting 1 > planting 3.

%factor 1 pedigree p=0.00097; N111AxChinese>N98, N110B; N109AxChinese>N98.

%factor 1 interactions p=0.04776; N110B (planting 3) < many other cultivars.

%factor 2 planting p=0.02495; no Tukey.

%factor 3 planting p=0.03718; planting 3 > planting 1.

%factor 3 interactions p=0.02428; N110B (planting 3) < N109AxDale (planting 2), N98 (planting 3), N109AxDale (planting 3); N98(3) > N98(2).

**References**

Broadhead, D.M., Coleman, O.H., 1973. Registration of Dale sweet sorghum. Crop Sci. 13, 776.

Gorz, H.J., Haskins, F.A., Johnson, B.E., 1990. Registration of 15 germplasm lines of grain sorghum and sweet sorghum. Crop Sci. 30, 762–763.

Knoll, J.E., Anderson, W.F., 2016. Yield components in hybrid versus inbred sweet sorghum. Crop Sci. 56, 2638-2646.

Rosenow, D.T., Dahlberg, J.A., Stephens, J.C., Miller, F.R., Barnes, D.K., Peterson, G.C., Johnson, J.W., Schertz, K.F., 1997. Registration of 63 converted sorghum germplasm lines from the Sorghum Conversion Program. Crop Sci. 37, 1399-1400.

Uchimiya, M., Knoll, J.E., Anderson, W.F., Harris-Shultz, K.R., 2017a. Chemical analysis of fermentable sugars and secondary products in 23 sweet sorghum cultivars. J. Agr. Food Chem. 65, 7629-7637.

Uchimiya, M., Knoll, J.E., Harris-Shultz, K.R., 2017b. Electrochemical evaluation of sweet sorghum fermentable sugar bioenergy feedstock. ACS Sustainable Chem. Eng. 5, 7352−7364.

Ugliano, M., 2016. Rapid fingerprinting of white wine oxidizable fraction and classification of white wines using disposable screen printed sensors and derivative voltammetry. Food Chem. 212, 837-843.

Wang, M.L., Zhu, C., Barkley, N.A., Chen, Z., Erpelding, J.E., Murray, S.C., Tuinstra, M.R., Tesso, T., Pederson, G.A., Yu, J., 2009. Genetic diversity and population structure analysis of accessions in the US historic sweet sorghum collection. Theoretical and Applied Genetics 120, 13-23.

Wu, X., Staggenborg, S., Propheter, J.L., Rooney, W.L., Yu, J., Wang, D., 2010. Features of sweet sorghum juice and their performance in ethanol fermentation. Ind. Crop. Prod. 31, 164-170.
